# Effects of Training Sets Sequence on Swimming Performance, Training Load and Physiological Responses

**DOI:** 10.3390/sports11120240

**Published:** 2023-12-04

**Authors:** Ioannis S. Nikitakis, Gregory C. Bogdanis, Giorgos P. Paradisis, Argyris G. Toubekis

**Affiliations:** 1 Division of Aquatic Sports, School of Physical Education and Sports Science, National and Kapodistrian University of Athens, 17237 Athens, Greece; inikitak@phed.uoa.gr; 2 Sports Performance Laboratory, School of Physical Education and Sports Science, National and Kapodistrian University of Athens, 17237 Athens, Greece; gbogdanis@phed.uoa.gr (G.C.B.); gparadi@phed.uoa.gr (G.P.P.)

**Keywords:** swimming, performance, interval training, heart rate variability, training load

## Abstract

The study examined the effect of set sequence on performance and physiological responses in a training session and in each set separately. Twelve male swimmers performed four sessions in a randomized order, including a combination of two training sets: (i) set A-set C, (ii) set C-set A, (iii) set B-set C, (iv) set C-set B. Set A consisted of 8 × 200 m at a speed corresponding to lactate threshold (30 s recovery), set B included 8 × 100 m at the maximal aerobic speed (30 s recovery), set C included 8 × 50 m sprints at 95% of the maximum 50 m speed (30 s recovery). Speed, blood lactate, pH, base excess, bicarbonate and heart rate variability (HRV) were measured. Speed in each set was similar between sessions irrespective of set sequence (*p* > 0.05). Physiological responses during sets A and C were similar in all sessions (*p* > 0.05). In set B, when applied after set C, the metabolic response increased, and HRV decreased (*p* < 0.05). Overall, session biochemical disturbance was higher when set C was applied before sets A and B (*p* < 0.05). The magnitude of metabolic and HRV responses in a set conducted at maximal aerobic speed, but not at lactate threshold intensity, is increased when applied after sprint intervals.

## 1. Introduction

Combining high-intensity and low-intensity training sets represents an important component of planning training sessions to enhance athletic performance [1]. Especially during the specific and competitive mesocycles of an annual training plan, the combination of training sets performed at different intensity domains in the same training session is a common practice [2]. During training sets around the lactate threshold (LT), blood lactate (BL) and oxygen uptake can be maintained stable at a range of 3–6 mmol·L^−1^ and 80–87% of VO_2_max, respectively, in competitive swimmers [3,4]. Accordingly, during training at intensities near the maximal aerobic speed (MAS), a continuous rise in lactate concentration may be observed, and BL values of 9–10 mmol·L^−1^ may be reached [5]. During repeated sprints (efforts lasting 10–30 s), a peak metabolic response (BL: 12–17 mmol·L^−1^) is expected due to the maximal exercise intensity [6,7].

When more than one training set is applied in a training session, apart from the acute physiological responses, the changes in physiological parameters during recovery after the first set may also influence performance and physiological responses observed during the following single or repeated sprints. Even after a six-minute recovery between sets, BL is maintained at values similar to those reached in the previous 8 × 25 m all-out training set (10–13 mmol·L^−1^), without affecting performance in a subsequent 50-m all-out effort [8]. When longer recovery (i.e., 10 min) is applied after a 200 m all-out effort, lactate concentration remains high (~7 mmol·L^−1^) with passive recovery and performance is reduced in a subsequent effort [9].

In a series of maximum-intensity efforts where glycolysis is activated, increased lactate and the resulting pH reduction inhibit glycolytic enzymes [10], leading to impaired muscle function. However, a moderate reduction in pH, as occurs in priming exercise, results in beneficial responses, such as faster oxygen uptake kinetics, increased muscle oxygen delivery and the activation of aerobic key enzymes [11,12]. A combination of the above-mentioned responses may enhance performance or alter the energy contribution in aerobic-dominated training sets when applied after high-intensity and short-duration efforts [12], thus affecting the observed physiological responses. On the other hand, an aerobic-dominated priming exercise (such as a long-duration training set) may induce a substantial reduction in glycogen stores [13] and magnify any perceptual response. However, the effects of aerobic priming on subsequent repeated efforts requiring increased anaerobic contribution have received limited attention in swimming.

A previous study examined the effect of aerobic and anaerobic set sequences in a training session, including an all-out training set of 4 × 50 m front crawl swimming [14]. However, the long 2 min resting interval applied between 50 m sprints in the previous study may substantially alter the acute physiological responses [15] compared to a short rest interval high-intensity training set that may regularly be applied in other types of training sets aiming to improve anaerobic capacity (speed endurance) [7]. When long recovery periods are applied between 50 m sprints (work:rest ratio >1:3) BL may reach levels above 18 mmol·L^−1^ [16,17] compared with training sets applied with shorter recovery periods (about 14 mmol·L^−1^ in 1:1 work:rest ratio [7,14]). The difference in work:rest ratios during sprint intervals may also be a significant cofactor affecting autonomic nervous system (ANS) function [18] and the glycolytic contribution in energy turnover affecting performance in subsequent effort [19].

Undoubtedly, there are training periods when it is required to incorporate two or more divergent training modalities, such as aerobic, speed and speed endurance training in the same session [20]. What remains unclear is whether an implementation of an aerobic-dominated training set may affect performance and physiological responses to a subsequent training set aiming to maintain anaerobic stimulus and vice versa. Thus, the purpose of this study was to examine the performance and physiological responses of aerobic sets and an anaerobic set separately and during a training session following different set sequences. It was hypothesized that a training session including sprint intervals followed by a training set that stimulates mainly aerobic metabolism would magnify the overall session metabolic and biochemical response compared to the reverse order. A secondary hypothesis was that performance and physiological responses in each set separately would be affected by the application of the previous training set.

## 2. Materials and Methods

Twelve male highly trained/national level swimmers (age: 19 ± 2 years, body mass: 80.2 ± 9.0 kg, height: 183 ± 6 cm) specialized in 100 and 200 m events completed this study. Their best performance in the 200 m front crawl was above the 90th percentile of the national record and 86th percentile of the world record (119.1 ± 5.8 s, 635 ± 91 World Aquatics points) [21]. All tests were carried out during the mesocycle of specific preparation and applied approximately the same time of day, each one separated by at least 48 h, in a 25 m indoor swimming pool with constant water and ambient temperature (24–25 °C, 27–28 °C, respectively). Participants recorded their diet two days before the first testing session and were asked to follow the same diet two days before each of the following testing sessions.

### 2.1. Preliminary Tests

Before each test, a standardized warm-up was applied (400 m front crawl, 200 m front crawl drills, 4 × 50 m front crawl at pace 80% of personal best 400 m, one 12.5 m sprint). On the first visit, swimmers performed 50 and 400 m front crawl all-out efforts applied with push-off from the pool wall. A 10-minute active and 20-minute passive recovery was allowed between efforts. The mean speed of the 400 m test was used for the assessment of MAS [22]. On a second visit, an incremental 5 × 200 m front-crawl test was performed and was used to determine the individual LT [23]. The time for each 200 m was recorded using a digital stopwatch (FINIS 3X300, Finis Inc., Livermore, CA, USA) by two independent timekeepers. Fingertip blood samples were collected within the first 30 s of recovery after each 200 m repetition to measure lactate concentration (Lactate Scout+, SensLab GmbH, Leipzig, Germany). Heart rate (HR) was recorded continuously using an optical HR sensor (OH1; Polar Electro Oy, Kempele, Finland).

### 2.2. Main Tests

According to performance times in preliminary testing, three training sets were planned with the following characteristics. Set A consisted of eight repetitions of 200 m (8 × 200 m) with intensity corresponding to the speed at LT [3]. Set B consisted of eight repetitions of 100 m (8 × 100 m) with intensity corresponding to MAS [24]. Both set A and B repetitions of 200 and 100 m were separated by a 30 s recovery period. The third set, C, consisted of eight repetitions of 50 m (8 × 50 m) swimming at 95% of the all-out 50 m effort conducted in the preliminary tests and 1:1 swimming:recovery ratio [7]. Four sessions including the above-mentioned training sets were performed in randomized order: (i) set A of 8 × 200 m followed by set C of 8 × 50 m (A-C), (ii) set C of 8 × 50 m followed by set A of 8 × 200 m (C-A), (iii) set B of 8 × 100 m followed by set C of 8 × 50 m (B-C), (iv) set C of 8 × 50 m followed by set B of 8 × 100 m (C-B). In each session, 10 min of passive recovery were applied between sets.

### 2.3. Measurements

Before and after each training set, a blood sample (90 μL) was collected in plastic heparinized tubes (Sarstedt AG & Co., Nümbrecht, Germany) from a pre-warmed finger and analyzed using an iSTAT biochemical analyzer (iSTAT Corporation, Princeton, NJ, USA) for the determination of pH in the blood and the calculation of base excess (BE) and bicarbonate (HCO_3_) concentration. Lactate concentration was measured before, at the middle and at the end of each set in a fingertip capillary blood sample (Lactate Scout+, SensLab GmbH, Leipzig, Germany). Delta values (Δ) between time points of measurements (post-set vs. pre-set) were calculated in all measured variables. The time to complete each swimming repetition was recorded to calculate the corresponding average speed. The fatigue index of set C was calculated: (highest speed-lowest speed) × 100/highest speed. The rating of perceived exertion (RPE, 0–10 scale) was recorded after each repetition, while HR was continuously monitored.

Overall session RPE (sRPE) was recorded 30 min after the completion of each session and was used to calculate subjective training load (TL) by multiplying sRPE by the duration of the training session in min [25]. The individualized training impulse was calculated for each set and for the total session to estimate the training load objectively (TRIMPi) [26]. For the entire session TRIMPi calculation, the average HR from the start of the first set until 10 min after the second training set was used, including HR in the recovery period between the two sets. In each session, heart rate variability (HRV) was recorded before the first set and after each set in a sitting position for 5 min using an electrocardiographic chest-strap HR monitor (H10 sensor, Polar Electro, Kempele, Finland), paired with a freely available smartphone application (Elite HRV, Asheville, NC, USA).

The night after each session, R–R intervals were recorded, and nocturnal HRV was calculated by analyzing a four-hour data set, excluding the first 30 min after going to bed [27]. The natural logarithm of root mean square successive difference (LnRMSSD) was used to examine the effect of each set, of each session and nocturnal HRV on ANS [28] to ensure the normality of distribution. All R–R files were exported from the Elite HRV smartphone application and stored on a separate computer for analysis using Kubios HRV 3.4.1 (Kuopio, Finland). Each file was corrected for ectopic beats and artifacts before analysis using a very strong level of artifact correction provided in Kubios HRV (R–R intervals that are larger/smaller than 0.05 s compared to the local average).

### 2.4. Statistical Analysis

Statistica v.10 software (Stat-Soft Inc., Tulsa, OK, USA) was used for data analysis. Sphericity was verified using Mauchly’s test. A two-way analysis of variance for repeated measures was used to examine differences in speed and physiological responses. A Tukey’s honest significant difference post hoc test was used to identify differences between means. Differences between sessions in entire session TRIMPi, nocturnal LnRMSSD, sRPE and TL were examined using dependent samples t-test. Cohen’s d effect size (ES) was calculated and was categorized as small (0.20–0.49), medium (0.50–0.79), and large (>0.80) [29]. A priori power analysis indicated a required sample size of n = 11, given error probability (0.05), power (0.84) and a medium effect size [30]. Considering the sample size in the present study (n = 12) and the corresponding partial eta-squared (η^2^ = 0.28), the calculated power of analysis corresponded to 0.97 [30]. Significance was set at *p* ≤ 0.05. Data are presented as mean ± SD.

## 3. Results

Mean speed in each set A, B or C was not influenced by the set sequence (*p* > 0.05, Table 1). Swimming speed in set C corresponded to 90–92% of the maximum 50 m speed instead of 95%, with no difference between sessions (*p* > 0.05). Furthermore, the fatigue index calculated in set C did not differ in both applied sequences (*p* > 0.05). There was no main effect between sessions in HR (*p* > 0.05). Heart rate in each set separately did not differ between A-C and C-A, but it was higher in set B of C-B compared to the B-C session (*p* < 0.05, Table 1). TRIMPi in set A was higher in C-A compared to the A-C session (*p* < 0.05, Table 1). Entire C-A TRIMPi was higher compared to A-C session (82.5 ± 30.0 vs. 66.8 ± 36.0 a.u., ES = 0.48, *p* < 0.05) while there was no difference between B-C and C-B session (B-C: 40.5 ± 17.5 vs. C-B: 46.5 ± 18.6 a.u., ES = 0.33, *p* > 0.05). However, TL (the product of sRPE by time) was not different between A-C and C-A as well as between B-C and C-B sessions (A-C: 234.4 ± 30.7, C-A: 239.9 ± 53.8 a.u., ES = 0.13, B-C: 147.1 ± 39.2, C-B: 159.9 ± 31.1 a.u., ES = 0.27, *p* > 0.05).

**Figure 1:**
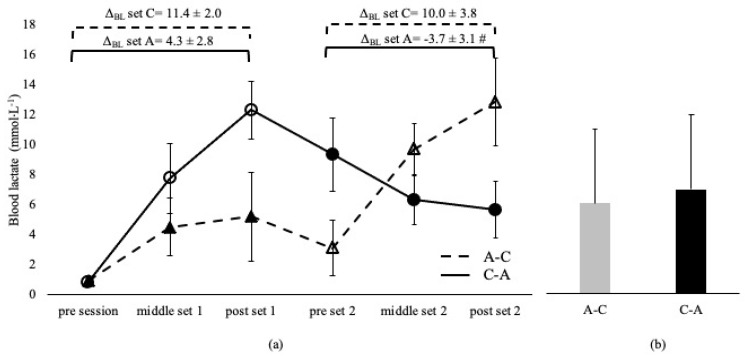
Blood lactate changes during A-C and C-A sessions (**a**) and average concentration of the entire sessions (**b**). Dashed lines and triangles indicate the A-C session, and continuous lines and circles indicate the C-A session. Open circles and triangles indicate set C, and filled circles and triangles indicate set A. Set A: 8 × 200 m with intensity corresponding to lactate threshold. Set C: 8 × 50 m at speed corresponding to 95% of 50 m maximum speed. Δ_BL_ set A: change from pre- to post-set A. Δ_BL_ set C: change from pre- to post-set C. #: *p* < 0.05 between sessions.

**Figure 2:**
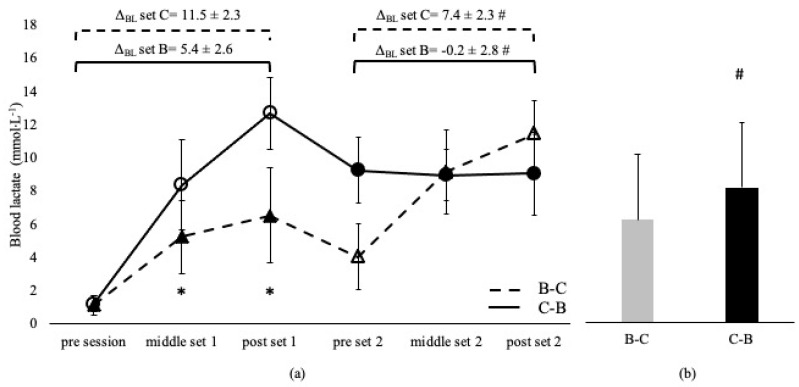
Blood lactate changes during Β-C and C-Β sessions (**a**) and average concentration of the entire sessions (**b**). Dashed lines and triangles indicate the Β-C session, and continuous lines and circles indicate the C-Β session. Open circles and triangles indicate set C, and filled circles and triangles indicate set Β. Set B: 8 × 100 m at maximal aerobic speed. Set C: 8 × 50 m at speed corresponding to 95% of 50 m maximum speed. Δ_BL_ set B: change from pre- to post-set B. Δ_BL_ set C: change from pre- to post-set C. *: *p* < 0.05 compared to the middle and post-set 2 in the C-B session. #: *p* < 0.05 between sessions.

Blood lactate was not different between sessions and between the corresponding sets, irrespective of the applied order in the A-C and C-A sessions (*p* > 0.05, Figure 1). BL was higher in C-B compared to the B-C overall session (*p* < 0.05) and at middle and post set B in C-B compared to the B-C session (*p* < 0.05). However, BL at set C was independent of the applied sequence (*p* > 0.05, Figure 2). Δ_BL_ at set A and B in A-C and B-C sessions are presented in Figure 1 and Figure 2. Δ_BL_ during set C was lower in B-C compared to the C-B session (Figure 2).

The measured pH in the A-C session was higher compared to C-A, and BE and HCO_3_ were lower in C-A compared to the A-C session (*p* < 0.05, Table 2). Nevertheless, set A or set C separately showed similar responses independent of the sequence in which they were applied (*p* > 0.05, Table 2). Regarding B-C and C-B sessions, the mean pH in the former one remained higher compared to the latter one (*p* < 0.05), while BE and HCO_3_ were lower in the C-B compared to the B-C session (Table 3, *p* < 0.05). Nevertheless, set B or set C separately showed similar responses independent of the applied sequence (Table 3, *p* > 0.05). Delta values in pH, BE, and HCO_3_ at sets A and B in all sessions are shown in Table 2 and Table 3.

The mean RPE in each set separately did not differ depending on the applied sequence (Table 2 and Table 3, *p* > 0.05). However, RPE at the start of sets A and B was higher in sessions C-A and C-B compared to the reverse sequence (Table 2 and Table 3, *p* < 0.05). LnRMSSD after set B in C-B session was lower compared to B-C session (*p* < 0.05, Table 3), while nocturnal LnRMSSD did not differ between sessions (A-C: 3.3 ± 0.2, C-A: 3.3 ± 0.2 ms, ES = 0.16, *p* > 0.05, B-C: 3.3 ± 0.1, C-B: 3.2 ± 0.1 ms, ES = 0.59, *p* > 0.05).

## 4. Discussion

The aim of the study was to investigate the effect of different sequences of training sets at LT (8 × 200 m), MAS (8 × 100 m) and sprint interval set of 8 × 50 m during a training session on performance and physiological responses. The present findings indicate that (i) performance in all sets, as evidenced by the ability to maintain the required speed, was not affected by the applied sets sequence, (ii) the metabolic response to an aerobic-dominated training set conducted in MAS was higher when applied after the 8 × 50 m set, (iii) acid-base balance in each set separately was not affected by the sets sequence. Interestingly, the metabolic acidosis of the entire training session was overall higher when the 8 × 50 m set was applied before the 8 × 200 and 8 × 100 m sets A and B, respectively, compared to the reverse order.

The observed results of BL following set C of 8 × 50 m are not in line with previous studies reporting lower values, which is probably attributed to age and level variation of swimmers (~14 mmol·L^−1^ vs. 10–11 mmol·L^−1^) [6]. Concerning BL's effects on performance, there is evidence suggesting that increased BL following set C is not related to fatigue during repeated sprints [31,32]. However, hydrogen ions produced in glycolysis may act to impair muscle function and, thus, performance [33]. The latter was not observed in sets A and B following set C as swimmers in the current and in a previous study [14] managed to maintain the required speed during sets applied in moderate (LT—set A) and severe (MAS—set B) swimming intensity domain.

Interestingly, swimmers performed set B of 8 × 100 m in the C-B session with higher lactate as opposed to the session started with the aerobic-dominated set at MAS (set B). Underlying mechanisms explaining the ability to maintain the pre-defined intensity of the aerobic-dominated training sets following repeated sprints are the faster oxygen uptake kinetics and the lower oxygen deficit, especially during the initial phase of each repetition [34]. The possible reduction of dependence on anaerobic metabolism may also be indicated by the reduced changes in BL at C-A and C-B sessions compared to the reverse order [19]. The negative and zero values of Δ_BL_ during sets A and B, when applied after the 8 × 50 m set C, may reflect a lower activation of glycolysis accompanied by higher activation of oxidative metabolism compared to reverse order sessions. The present data suggest that lactate was removed during set A, reflecting the lower glycolytic contribution to energy demand when training at intensities lower than MAS [35]. Moreover, the lower Δ_BL_ in set C during the B-C session may also reflect a lower glycolytic contribution compared to the reverse order, which may be attributed to the already high BL caused by the implementation of the preceded set B. On the other hand, set B requires higher activation of glycolysis compared to set A [35]; thus, maintenance of BL at higher values seems reasonable in the C-B session.

The maintenance of lactate concentration during the following set B resulted in an overall higher mean BL of the C-B session compared to the reverse order. The longer the time spent with high BL, the greater the impact on biochemical indices. Indeed, indices that are involved in acid-base balance (pH, BE and HCO_3_) declined after set C, reaching values similar to previous reports (pH of ~7.2) [36] and remained low until swimmers started to perform the second set A or B. When aerobic-dominated sets are performed at the start of the session, the metabolic perturbation is lower than that caused by set C, and a significant reduction in metabolite concentration takes place after the 10 min resting interval period. In such sessions (A-C and B-C), a high metabolic disturbance occurs at the last part of the session, resulting in a lower overall physiological impact compared to reverse order. Whatever the case, the acid-base balance response in each set was independent of the applied set sequence. The modest reduction in pH caused by the preceding set B of 8 × 100 m did not alter the ability of swimmers to maintain the required speed in the subsequent set C of 8 × 50 m. In a previous study, we observed maintained performance at 95–97% of 50 m maximum in a 4 × 50 m training set, however, with a long 2 min resting interval [14]. Surprisingly, despite the short resting interval of 30 s and the extended number of repetitions in the current study, forcing swimmers to be exposed to low pH for a longer period, they were able to maintain speed at 90–92% of their 50 m maximum. It is likely that in this group of well-trained swimmers, the 10 min period of passive resting interval applied after set B was adequate to preserve performance in the latter set [31].

Concerning the cardiac responses and given that HR may express the energy cost during submaximal exercise [37], it seems that when set C is performed first, the energy demand was increased in the subsequent set B. Despite the fact that HR is a less valid indicator of internal load in short-duration efforts, TRIMPi can be used to monitor this parameter [38,39]. Set A, when applied after sprint intervals of 8 × 50 m of set C, induced a higher internal load compared to the reverse order. In C-A session, the preceded sprints may act as a priming training set [12], leading to enhancement of the already activated metabolism at the start of the following set A. In that case, HR may respond faster at the initial repetitions, reaching average values more quickly compared to the A-C session. Despite the higher TRIMPi in set A during the C-A session, swimmers did not show higher perceived effort to maintain the required speed in each set, as the RPE in each set did not differ between sessions. Despite the fact that subjective tools (e.g., RPE) are convenient and valid for monitoring training load, sRPE can usually be misused by athletes mainly because of limited familiarization of the latter with such a tool [39]. In that case, an objective form of monitoring (e.g., TRIMP) may be more appropriate. The entire C-A session TRIMPi was higher compared to the reverse order. The latter may be attributed to the relatively higher cardiac response (HR) during set A in the C-A session, as evidenced by the corresponding medium effect size between sessions. The different effect on the induced TRIMPi, but not sRPE, as a result of the sets’ applied sequence, highlights a novel observation of the current study. Coaches should take into account this new information as the systematic implementation of training sessions that cause high internal load may affect long-term performance [40].

The effect of the MAS training set on ANS seems to be dependent on the applied set sequence when combined with sprint intervals. Specifically, a parasympathetic withdrawal was observed after the first training set as expected, but the ANS disturbance in set B was higher when the 8 × 50 m set C preceded. This is a novel finding not observed in a previous study in which the anaerobic-dominated training set was conducted with a 1:4 work:rest ratio, inducing higher metabolic acidosis [14]. In fact, the magnitude of metabolic acidosis may not be the single critical factor for ANS response, but the duration of recovery periods between repetitions may also be another issue leading to high parasympathetic withdrawal [18]. These may explain parasympathetic withdrawal maintenance during the subsequent set conducted in MAS. Moreover, the metabolic acidosis caused by set C was maintained during the following set B (as evidenced in the present study) while was attenuated during the same training set of 8 × 100 m conducted in MAS in a previous study [14]. Thus, the resting interval and set duration may lead to the maintenance of ANS disturbance at higher levels compared to the session which started with a lower-intensity training set (MAS - 8 × 100 m). The overall potential perturbation of cardiac parasympathetic activity after each session was recorded using HRV data during night sleep after each training session. Considering the limitation arising from the fact that baseline HRV was not recorded the night before each session, a parasympathetic reactivation occurred after each session, which reflects wellness and readiness to perform [28], and the magnitude of parasympathetic reactivation was similar between sessions independent on the applied sequence. Nevertheless, the LnRMSSD selection as an index of HRV and the very strong filter used for the analysis may be considered limitations of the present study.

## 5. Conclusions

The applied sequence of training sets does not induce any negative impact on set performance time in highly trained swimmers. Training sessions including sprint intervals of 8 × 50 m as a first set, increase the magnitude of metabolic, cardiac and ANS disturbance of subsequent aerobic-dominated training set B performed at MAS but not at LT. In contrast, the physiological responses to sprint intervals are unaltered by set sequence. The overall session biochemical disturbance, caused when sprint intervals are applied before an aerobic-dominated training set, may induce a higher internal training load in swimmers, and this is one of the novel findings in the current study.

## Figures and Tables

**Table 1:** Swimming speed, heart rate (HR), and individualized training impulse (TRIMPi) in each set A, B, and C were applied in four sessions of the present study (sessions A-C, C-A, B-C, C-B). Effect size (ES) for the comparison between the same sets. *: *p* < 0.05 between the same training sets in each sequence. Set A: 8 × 200 m with intensity corresponding to lactate threshold. Set B: 8 × 100 m at maximal aerobic speed. Set C: 8 × 50 m at speed corresponding to 95% of 50 m maximum speed.

	Session A-C		Session C-A			
	set A	set C	set C	set A	ES set C	ES set A
Speed (m·s^−1^)	1.43 ± 0.08	1.71 ± 0.08	1.72 ± 0.06	1.42 ± 0.07	0.08	0.21
HR (beats·min^−1^)	173 ± 12	180 ± 6	177 ± 4	177 ± 6	0.60	0.63
TRIMPi (a.u.)	63.6 ± 40.4	18.0 ± 7.7	16.4 ± 6.2	82.0 ± 45.0 *	0.23	0.43
	Session B-C		Session C-B			
	set B	set C	set C	set B	ES set C	ES set B
Speed (m·s^−1^)	1.53 ± 0.06	1.71 ± 0.07	1.71 ± 0.06	1.53 ± 0.07	0.02	0.03
HR (beats·min^−1^)	173 ± 11	179 ± 7	176 ± 6	178 ± 6 *	0.52	0.61
TRIMPi (a.u.)	34.2 ± 20.8	19.1 ± 9.1	16.4 ± 5.3	39.4 ± 14.9	0.37	0.29

**Table 2:** Blood pH, base excess (BE) and bicarbonate (HCO_3_), the natural logarithm of root mean square successive difference (LnRMSSD) and rate of perceived exertion (RPE) in sets A and C within the two experimental sessions of the study (sessions A-C, C-A). Effect size (ES) between sessions. *: *p* < 0.05 between the same training set in each session. #: *p* < 0.05 between sessions. Set A: 8 × 200 m with intensity corresponding to lactate threshold. Set C: 8 × 50 m at speed corresponding to 95% of 50 m maximum speed. Δ_set A_: change from pre- to post-set A, Δ_set C_: change from pre- to post-set C.

		Session A-C								Session C-A								
		Pre-Set A	Post-Set A	Δ_set A_	Pre-Set C	Post-Set C	Δ_set C_	Overall Session		Pre-Set C	Post-Set C	Δ_set C_	Pre-Set A	Post-Set A	Δ_set A_	Overall Session		ES
pH		7.42 ± 0.02	7.37 ± 0.05	−0.05 ± 0.05	7.38 ± 0.04	7.23 ± 0.05	−0.15 ± 0.07	7.35 ± 0.08		7.42 ± 0.01	7.20 ± 0.05	−0.22 ± 0.05	7.25 ± 0.07	7.36 ± 0.03	0.11 ± 0.08 *	7.31 ± 0.10 **^#^**		0.45
BE (mmol·L^−1^)		1.8 ± 2.0	−4.4 ± 4.7	−6.3 ± 4.0	−3.2 ± 3.9	−14.6 ± 3.7	−11.4 ± 5.2	−5.1 ± 7.0		0.9 ± 1.4	−15.6 ± 2.5	−16.5 ± 2.3 *	−13.3 ± 3.9	−5.7 ± 2.3	7.6 ± 4.0 *	−8.4 ± 7.1 **^#^**		0.47
HCO_3_ (mmol·L^−1^)		26.1 ± 1.8	21.0 ± 3.9	−5.1 ± 3.2	22.2 ± 3.3	12.9 ± 2.7	−9.3 ± 4.0	20.5 ± 5.7		25.3 ± 1.3	12.5 ± 1.8	−12.8 ± 1.7*	13.9 ± 2.7	19.8 ± 1.9	5.9 ± 2.8 *	17.9 ± 5.5 **^#^**		0.48
Ln RMSSD (ms)		2.7 ± 0.3	2.0 ± 0.4	-	-	1.9 ± 0.4	-	2.2 ± 0.5		2.7 ± 0.2	1.7 ± 0.4	-	-	1.8 ± 0.4	-	2.1 ± 0.6		0.24
RPE (a.u.)		1.8 ± 1.7	6.1 ± 2.2	4.3 ± 1.7	4.5 ± 2.6	9.2 ± 1.1	4.7 ± 2.8	7.9 ± 0.9		2.5 ± 2.2*	8.8 ± 1.5	6.9 ± 2.0	4.4 ± 2.3 *	7.0 ± 2.6	2.7 ± 2.7	8.1 ± 1.9		0.12

**Table 3:** Blood pH, base excess (BE) and bicarbonate (HCO_3_), the natural logarithm of root mean square successive difference (LnRMSSD) and rate of perceived exertion (RPE) in sets B and C within the two experimental sessions of the study (sessions B-C, C-B). Effect size (ES) between sessions. *: *p* < 0.05 between the same training set in each session. #: *p* < 0.05 between sessions. Set B: 8 × 100 m at maximal aerobic speed. Set C: 8 × 50 m at speed corresponding to 95% of 50 m maximum speed. Δ_set B_: change from pre- to post-set B, Δ_set C_: change from pre- to post-set C.

		Session B-C								Session C-B								
		Pre-Set B	Post-Set B	Δ_set B_	Pre-Set C	Post-Set C	Δ_set C_	Overall Session		Pre-Set C	Post-Set C	Δ_set C_	Pre-Set B	Post-Set B	Δ_set B_	Overall Session		ES
pH		7.40 ± 0.04	7.34 ± 0.05	−0.06 ± 0.07	7.36 ± 0.04	7.24 ± 0.05	−0.12 ± 0.06	7.34 ± 0.07		7.41 ± 0.02	7.22 ± 0.04	−0.19 ± 0.04 *	7.26 ± 0.04	7.32 ± 0.04	0.06 ± 0.06 *	7.30 ± 0.08 ^#^		0.43
BE (mmol·L^−1^)		0.3 ± 1.2	−7.2 ± 4.0	−7.5 ± 3.9	−5.1 ± 3.0	−14.3 ± 2.8	−9.3 ± 3.7	−6.6 ± 6.0		0.5 ± 1.9	−14.9 ± 2.0	−15.4 ± 2.6 *	−12.9 ± 2.5	−9.4 ± 2.7	3.5 ± 3.4 *	−9.2 ± 6.4 ^#^		0.42
HCO_3_ (mmol·L^−1^)		25.1 ± 0.8	18.6 ± 3.3	−6.5 ± 3.2	20.3 ± 2.5	13.1 ± 2.1	−7.2 ± 2.9	19.3 ± 4.9		25.1 ± 1.7	12.8 ± 1.4	−12.3 ± 2.1 *	14.2 ± 2.0	16.7 ± 2.1	2.5 ± 2.6 *	17.2 ± 5.1 ^#^		0.41
Ln RMSSD (ms)		2.9 ± 0.3	2.1 ± 0.4	-	-	1.9 ± 0.3	-	2.3 ± 0.5		2.7 ± 0.2	1.7 ± 0.4	-	-	1.8 ± 0.4 *	-	2.0 ± 0.6		0.50
RPE (a.u.)		2.4 ± 2.2	7.5 ± 2.2	5.0 ± 2.7	4.6 ± 1.6	9.4 ± 0.8	4.8 ± 1.9	7.5 ± 2.0		3.1 ± 2.4 *	8.9 ± 1.4	5.8 ± 2.6	5.1 ± 1.9 *	8.3 ± 1.6	3.3 ± 1.4	7.9 ± 1.1		0.30

## Data Availability

The data presented in this study are available on request from the corresponding author. The data are not publicly available due to privacy and ethical reasons.
